# Paper vs. screen: inclination matters during global–local visual processing

**DOI:** 10.3389/fpsyg.2025.1701298

**Published:** 2026-01-07

**Authors:** Paul Nibaudeau, Nicolas Poirel

**Affiliations:** 1 Université Paris Cité, CNRS, LaPsyDÉ, Paris, France; 2 GIP Cyceron, Campus Jules Horowitz, Caen, France; 3 Institut Universitaire de France (IUF), Paris, France

**Keywords:** digital media, global processing, inclination, local processing, screen vs. paper

## Abstract

The use of new technologies has opened the field to numerous studies regarding their impact on cognition. While many studies have suggested that paper presents an advantage over screens, the impact of the medium during local–global visual attentional processes has never been investigated. Visual processing is indeed characterized by a global advantage (i.e., faster global than local processing) and by global interference (i.e., interference from global information during local processing). We designed a task to assess whether global–local processing could be modulated by the type of medium. Owing to the frequent variations in paper and screen presentations encountered in daily life, we also studied differences in inclination between screen and paper. The results revealed an interaction between inclination and global–local processes. Global interference was more pronounced when the medium was tilted, whereas similar global and local interference effects were observed when the medium was flat. There was no effect of the medium *per se*. Taken together, these results suggest no evidence of an impact of medium during local–global visual processing but rather suggest an impact of the inclination. This factor is discussed and should be considered in future studies on topics regarding the impact of the digital environment on humans.

## 1 Introduction

Over the last few decades, the use of new technologies has become a major educational, health and social issue, and this topic is currently the source of many studies, debates, and discussions, particularly in psychological sciences within the domain of child development (e.g., McArthur et al., 2022; Madigan et al., 2019; Radesky and Christakis, 2016; see also Madigan et al., 2020; Muppalla et al., 2023, for a recent review). Millennials were born into a world where technology is omnipresent and will continue to live in such a world. Among the various studies examining the links between digital technologies and young people’s physical health (e.g., Davies et al., 2012; Domoff et al., 2019; Vizcaino et al., 2020), mental health (e.g., Camerini et al., 2022; Tang et al., 2021), cognitive development (e.g., Guellai et al., 2022) and education (e.g., Ozerbas and Erdogan, 2016), a growing number of studies have also examined the impact of screen use on cognitive functions. For instance, recent studies have focused on determining the difference between screen and paper on reading performance (Mangen et al., 2013; Margolin et al., 2013; Rockinson-Szapkiw et al., 2013). A meta-analysis conducted by Delgado et al. (2018) with more than 170,000 participants concluded that comprehension performance was better when reading on paper than when reading on screen, regardless of age (from 6 years of age to adult). These results were modulated by the type of task performed during reading (i.e., understanding an informative text seemed easier on paper than on screen, whereas a narrative text showed no medium effect) and by time constraints (i.e., the advantage of paper over screen was greater only when a time limit was imposed during reading). Halamish and Elbaz (2020) also addressed the question of the link between performance and media preference. They reported similar results to those cited above for text comprehension and further showed that these results were independent of children’s preference for a medium during reading activity. These findings are in line with Delgado et al., 2018 meta-analysis, which questions the hypothesis of a positive impact of digital tools *per se* on our reading comprehension with this type of medium. It is worth noting that although screen inferiority in reading comprehension has been suggested in several meta-analyses (Clinton, 2019; Delgado et al., 2018; Salmerón et al., 2023), other studies and meta-analyses have reported contrasting findings, showing no significant effect of the medium on reading comprehension (Delgado and Salmerón, 2022; Mangen et al., 2019; Xu et al., 2017). Alternatively, some of these studies have highlighted the impact of the type of device used during the task. Salmerón et al. (2023), for instance, reported that the screen inferiority effect was smaller with handheld devices than with computers during reading comprehension tasks. Overall, studies comparing reading on paper and on screen tend to show a slight and relatively consistent disadvantage of screen reading in terms of text comprehension and retention, particularly for long expository texts, under time constraints, or when reading involves continuous scrolling rather than stable pagination (see also Sparrow et al., 2011). In line with evidence that visual attention plays a key role in reading acquisition, several studies (Bosse and Valdois, 2009; Valdois et al., 2019, Delgado et al., 2018) interpret part of these effects in terms of attention and resource allocation (see also Clinton, 2019). It has also been suggested that screen readers tend to allocate less time and adopt a more superficial reading strategy, whereas paper readers favor a more stable and in-depth allocation of attention (Ackerman and Lauterman, 2012; Støle et al., 2020).

The impact of screen use on attention has also been investigated through video game studies, both in healthy individuals and in participants with attention disorders (e.g., ADHD, Beyens et al., 2018; Masi et al., 2021; Swing et al., 2010). Importantly, while this line of work provides valuable insights into how screen-based technologies may modulate attentional processes, it should not be conflated with the broader literature comparing reading supports (paper versus screen). The literature highlights both the potential risks of using (and especially overusing) video games in relation to the development of attentional deficits (Cardoso-Leite and Bavelier, 2018; Choi et al., 2020; Peñuelas-Calvo et al., 2022) and the benefits of video game training and use for attentional processes (Bioulac et al., 2008; Chan and Rabinowitz, 2006; Gentile et al., 2012; Russell and Newton, 2008; Swing et al., 2010). In this vein, digital training programs using action video games have been associated with improvements in visual attention abilities in non-clinical populations (Green and Bavelier, 2003; Schubert et al., 2015; Wilms et al., 2013). Although these effects may also be attributable to the specific demands of gameplay (e.g., immediate feedback, multitasking), a study by Martinez et al. (2023) that modeled the time spent playing either video games or board games, showed that only video games predicted higher performance in cognitive components such as mental flexibility, working memory, visuospatial processing, and fluid intelligence. The authors suggested that specific features of digital tools, such as dynamic interactive gameplay, might engage and train these cognitive components more effectively than board games.

Interestingly, another factor inherent to digital media *per se* appears to influence cognitive processing during screen-based activities. In particular, studies using tablets and computers have shown that the device’s angle affects performance (reading/pointing), indicating that performance seems sensitive to display orientation (Albin and McLoone, 2014; Young et al., 2012). Young et al. (2012) reported that extreme tablet angles were associated with slower and less accurate task performance, suggesting that suboptimal tilt increases both the attentional and oculomotor demands required to process visual information efficiently. This finding, consistent with that reported by Salmerón et al. (2023, see above) supports the view that when the medium is tilted, cognitive and motor responses may be delayed due to variations in the spatial reference frame (Bagust et al., 2013; Goodenough et al., 1979; Morgan et al., 2015). It therefore seems conceivable that attentional processes could be sensitive to the inclination of the medium, a potential effect that has never been examined in the context of screen versus paper use. Indeed, because screens and paper are rarely used in the same orientation (i.e., paper is usually flat, whereas screens are often inclined), the angle of presentation may also affect cognitive processes such as attention.

The present study was thus designed to examine the relationship between screen versus paper use, inclination, and the ability to process global and local information during an attentional task. In daily life, visual information is indeed processed hierarchically at both local and global levels, via selective attention (Serences and Kastner, 2014). The principle of the global precedence effect (GPE; Navon, 1977, 1981) implies that visual information is processed along a continuum from global structuring to finer local processing (Navon, 1977). In his study, Navon presented an experiment with compound stimuli, consisting of large global letters formed by a group of smaller local letters. Participants had to detect either the global target or local target, and the response times (RTs) revealed two components of the GPE. First, a global advantage effect, characterized by faster global processing than local processing. Second, a global-to-local interference effect, characterized by a stronger interference effect from global distractors during local processing than from local distractors during global processing (see also Gerlach and Poirel, 2018; Poirel et al., 2008a, 2008b, 2012, 2014). These effects were also highlighted during natural scene perception (e.g., Bar, 2004; Bullier, 2001; Kauffmann et al., 2014), and global–local processes were also shown to be linked to object visual processing in daily life (Gerlach and Poirel, 2018). Note that several studies have explicitly linked global–local processing to attentional mechanisms (e.g., Fredrickson and Branigan, 2005; Roux and Ceccaldi, 2001; Krakowski et al., 2015) and to cognitive control (e.g., Krakowski et al., 2016; Poirel et al., 2014). The study of global–local processes is also of direct practical interest, as it provides insight into how individuals allocate their attention to visual information across a wide variety of contexts (e.g., reading, learning at school, see, e.g., Krakowski et al., 2016) and, more generally, how human beings react to the visual world (see, e.g., Fredrickson and Branigan, 2005; Hegdé, 2008; Poirel et al., 2012).

To our knowledge, although some previous studies have investigated the impact of screens on attentional processes (e.g., Brasel and Gips, 2011), no study has examined the combined effect of medium (i.e., paper sheet vs. screen display) and inclination (flat vs. tilted, as is usually the case for paper and computer screen, respectively) on attentional processes during global–local processing. Using Navon’s classical paradigm with compound stimuli, we hypothesized faster global RTs than local RTs (i.e., global advantage) and faster RTs for neutral trials than for incongruent trials (i.e., compound stimuli composed of a letter at one level and a nonletter at the other level, minimizing interference, and compound stimuli composed of different large and small letters, respectively, see Poirel et al., 2008a, 2008b; Beaucousin et al., 2011), particularly during local tasks (i.e., global interference effect). In line with the literature on screen effects during reading (Delgado et al., 2018), we also expect slower RTs in the screen condition than in the paper condition, due to a potentially greater attentional efficiency when performing task on paper. This effect was expected to be associated with a stronger global interference effect in the screen condition than in the paper condition. The present study was also designed to explore the potential confounding variable of inclination when comparing paper and screen performance, given that a computer screen is typically inclined whereas a sheet of paper is usually flat on a desk. We therefore varied the inclination of the medium (screen and paper) and, in line with the results of Salmerón et al. (2023), we hypothesized that performance would be better under flat than under tilted conditions, regardless of the support.

## 2 Materials and methods

### 2.1 Participants

Sixty-eight healthy volunteer participants from the Institut de Psychologie of Université Paris Cité (20.22 ± 3.87 years, mean ± standard deviation) participated in the experiment. All participants provided written informed consent in accordance with the Declaration of Helsinki (BMJ 1991; 302:1194). The participants were randomly assigned to one of the two media (paper or screen) and to one of the two inclination conditions (tilted 110° or flat).

### 2.2 Instruments and procedure

**Figure 1:**
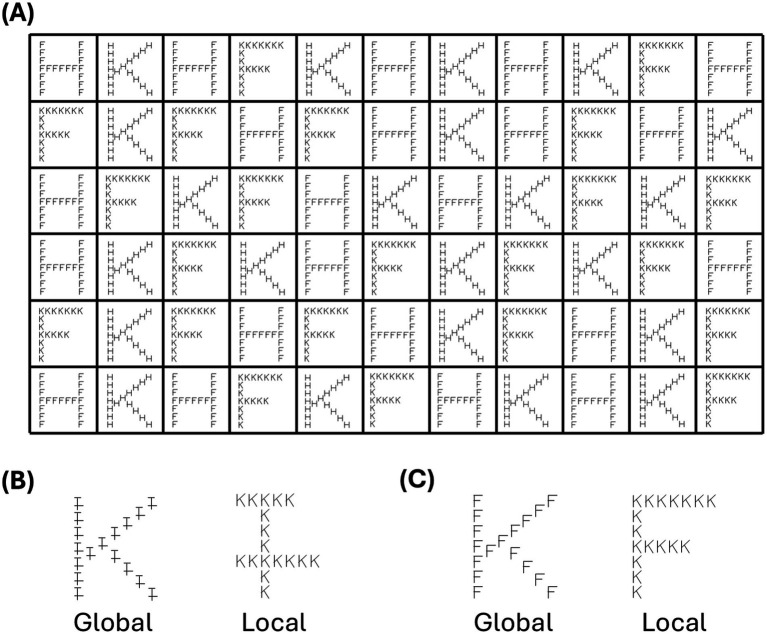
Presentations of the stimuli. The trials, an example of which is shown in interferent **(A)**, consist of 66 stimuli (6 rows and 11 columns), delimited by boxes. The stimuli can be neutral **(B)**, i.e., with a single nameable level (global or local), or interfering **(C)**, i.e., with two nameable levels (global and local).

For each group, participants had to name series of letters either at the global or local level of compound stimuli, from left to right and from top to bottom, according to the instructions (i.e., global or local conditions, see Figure 1A). The stimuli were randomly positioned in a grid, with 66 trials composed of 3 different letters and nonletters (Figures 1B,C) for each grid, spread over 6 rows and 11 columns. Given the black frame around the computer screen and the impact of a frame on hierarchical attentional processes (Bouhassoun et al., 2022), a black frame was also added to the paper version. Each grid was printed on an A4 white paper sheet (paper condition) or were presented via Microsoft PowerPoint© software (screen condition) on a personal laptop with a 15-in. screen. Four different conditions were tested using a between-subject design, in order to experimentally manipulate the type of support and its inclination. We used the same screen for the inclined condition as for the flat condition (instead of using two screens, such as a computer and a tablet). For the paper condition, stimuli were presented flat on the table or tilted, with the paper placed on the computer screen (device switched off). The tilt angle of 110° was maintained throughout for both screen and paper experimental conditions, as well as the participant’s distance from the apparatus, approximately 50 cm, so that the participant could be comfortable during the task. The instructions induced processing at the required hierarchical level, which could be local (i.e., participants had to name the small letters as quickly and as accurately as possible) or global (i.e., participants had to name the large letters as quickly and as accurately as possible; see Figure 1). After general instruction and a training session, each experimental condition was presented using the 4 grids of 66 trials each (i.e., local neutral, global neutral, local interference, and global interference). The order of the conditions presented was randomized between participants. The participants performed the task by naming each stimulus from left to right and from top to bottom, and RTs were recorded from the first denomination to the last item. RTs were then recorded for each condition, manually by the experimenter and reported for each participant, to the nearest tenth of a second. The experimenter had at his disposal a grid for reading the stimuli according to the experimental conditions, in order to check the veracity of the trials and reported corrected errors (i.e., the number of errors that were corrected instantaneously) and uncorrected errors for each trial.

### 2.3 Sample size determination

To evaluate the statistical validity of this analysis, we performed an *a priori* power analysis using G*Power 3.1 (Faul et al., 2007) with a 2 (level: global or local) × 2 (type of trial: neutral or incongruent) × 2 (media: paper or screen) × 2 (inclination: tilted or flat) mixed-design. The analysis indicated that a sample size of 36 participants (9 per group) was sufficient to detect a medium effect size (*f* = 0.25) with a power of 0.80 and an *α* level of 0.05. The *a priori* power analysis was conducted assuming a medium effect size, in line with Cohen’s (2013) conventions and with the small-to-medium effects typically reported in studies comparing paper and screen (Delgado et al., 2018) and studies using global–local visual-processing task (e.g., Gerlach and Poirel, 2020).

## 3 Results

Analyses were performed only on RTs (Table 1), given the floor effect observed on the number of corrected and uncorrected errors. All data and materials used within this study will be made available, upon request.

**Table 1:** Reaction times (in seconds, mean and standard deviation) according to experimental conditions.

Variable^a^		Paper				Screen			
		With inclinaison		Without inclinaison		With inclinaison		Without inclinaison	
		*M*	SD	*M*	SD	*M*	SD	*M*	SD
Trial type and level									
Neutral	Global	33.0	6.6	33.5	4.2	33.3	6.4	33.0	5.3
	Local	35.1	6.6	36.3	3.6	35.1	5.7	34.3	4.3
Interference	Global	35.0	6.9	38.6	6.7	37.3	8.1	38.9	6.4
	Local	41.5	7.7	40.9	5.1	42.3	7.3	38.9	5.4

^a^Within subject variables are included in rows, while between-subjects variables are included in columns. *N* = 68.

**Figure 2:**
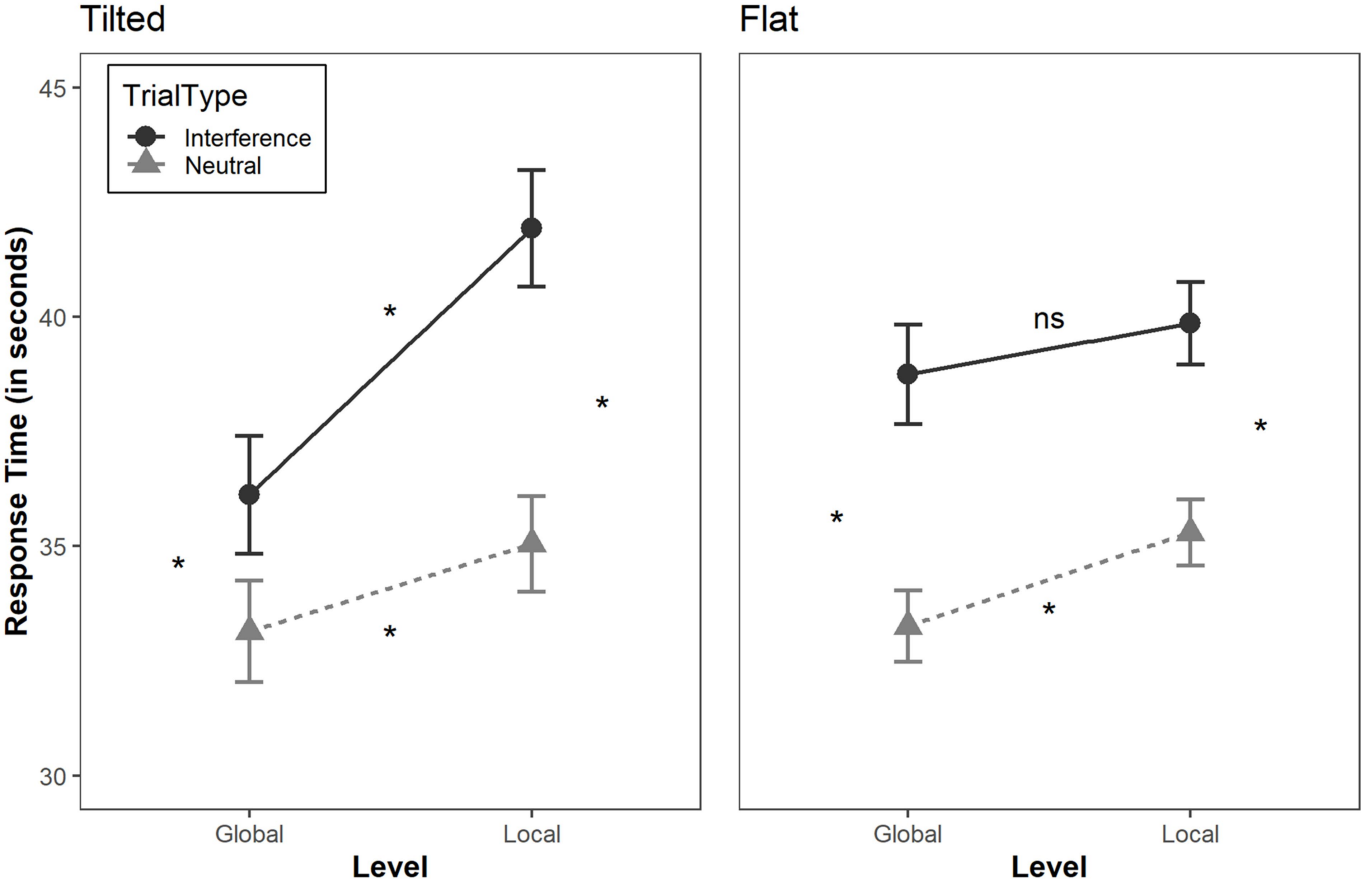
Response time (RTs) in seconds as function of trial type (Neutral or Interference), Level (Global or Local) and Inclination (Tilted or Flat). *ns* > 0.05, * < 0.05. Adjusted *t-test* with Bonferroni correction were performed and reported when comparing two conditions.

RTs were analyzed via R (R Core Team, 2022) with the package bruceR (Bao, 2023) to compute mixed-design ANOVA, and ggplot2 (Wickham, 2016) was used to visualize the data. We conducted 2 (level: global or local) × 2 (type of trial: neutral or incongruent) × 2 (media: paper or screen) × 2 (inclination: tilted or flat) mixed-design ANOVA (Table 2). We used an adjusted t test with Bonferroni correction when we compared two means. The four-way mixed-design ANOVA revealed a main effect of the level and type of trial. The participants processed global letters faster than local letters, *F* (1, 64) = 49.685, *p* < 0.001, *η^2^p* = 0.437, and they processed neutral trials faster than incongruent trials, *F* (1, 64) = 105.315, *p* < 0.001, *η^2^p* = 0.622. There was no main effect of media and no main effect of inclination (both *Fs* < 1). Inclination × Trial Type × Level interaction was not modulated by the media (*F* < 1), but we observed a significant inclination × trial type × level interaction, *F* (1, 64) = 15.948, *p* < 0.001, *η^2^p* = 0.199. On the one hand, the ANOVA on the tilted condition revealed a classic global advantage, *F* (1, 32) = 61.911, *p* < 0.001, *η^2^p* = 0.659 as well as a trial type x level interaction, *F* (1, 32) = 23.951, *p* < 0.001, *η^2^p* = 0.428. Participants were significantly more slowed down by the interfering letter in the local condition (i.e., global interference) than in the global condition (i.e., local interference) (local incongruent *minus* local neutral vs. global incongruent *minus* global neutral, *p* < 0.001). In contrast, the ANOVA on the flat condition also revealed a global advantage effect *F* (1, 32) = 7.055, *p* = 0.012, *η^2^p* = 0.181, as well as an interference characterized by a main effect of trial type, *F* (1, 32) = 54.509, *p* < 0.001, *η^2^p* = 0.630, but no trial type x level interaction. *F* (1, 32) = 1.031, *p* = 0.318, *η^2^p* = 0.031. In this case, global and local interference effects were similar (local incongruent *minus* local neutral vs. global incongruent *minus* global neutral, *p* > 0.05; see Figure 2). Finally, statistical analyses revealed small but statistically significant differences in global interference between tilted and flat conditions, *t* (66) = 2.12, *p* = 0.038, *d* = 0.51, and in local interference between flat and tilted conditions, *t* (66) = −2.13, *p* = 0.037, *d* = −0.52. Given the small effect sizes and the *p*-values being close to the 0.05 threshold, these results should be interpreted with caution.

**Table 2:** 2x2x2x2 mixed-ANOVA on reaction times (RTs).

Effect	Df1	Df2	MS	F	*η^2^p*	*p*
Media	1	64	0.418	0,004	0.000	0.953
Inclinaison	1	64	3.639	0,031	0.000	0.861
Media x Inclinaison	1	64	62.842	0,532	0.008	0.469
TrialType	1	64	1682.545	105,315	0.662	<0.001
Media x TrialType	1	64	13.297	0,832	0.013	0.365
Inclinaison x TrialType	1	64	0.163	0,010	0.000	0.920
Media x Inclinaison x TrialType	1	64	4.738	0,297	0.005	0.588
Level	1	64	502.928	49,685	0.437	<0.001
Media x Level	1	64	34.835	3,441	0.051	0.068
Inclinaison x Level	1	64	87.804	8,674	0.119	0.004
Media x Inclinaison x Level	1	64	4.275	0,422	0.007	0.518
TrialType x Level	1	64	37.489	6,092	0.087	0.016
Media x TrialType x Level	1	64	3.510	0,570	0.009	0.453
Inclinaison x TrialType x Level	1	64	98.136	15,948	0.199	<0.001
Media x Inclinaison x TrialType x Level	1	64	0.244	0,040	0.001	0.843

## 4 Discussion

In the present study, we compared different media and their inclination to investigate whether global–local visual-attentional processing varied according to screen, paper and orientation. Using a classic global–local task with a compound stimulus experiment, we replicated the well-known Global Precedence Effect (GPE, Navon, 1977; Poirel et al., 2008a), either with a screen or paper presentation. Interestingly, we did not observe an impact of the media (i.e., screen or paper), since no main effect or interaction including media was found on the RTs. Instead, the main result observed here suggests that inclination plays a key role during the visuo-attentional task. Indeed, as mentioned above, the classical GPE reported in the literature was observed when the medium was tilted (i.e., global information was processed faster than local information, and interference from global information was stronger than from local information, see Gerlach and Poirel, 2018), but not when the medium was flat. Indeed, in the flat condition, although a global advantage was observed (i.e., global information was processed faster than local information), we also found equivalent global-to-local and local-to-global interference effects. These results suggest that some aspects of participants’ visuo-attentional processes are modulated by inclination *per se*, but not by the medium. Serences and Kastner (2014) suggested that bottom-up stimulus-driven modulations are involved in the early processes of visual attention in primary brain areas such as the V1 region. It therefore seems conceivable that the inclination of the medium affected these brain regions and modulated the GPE, depending on the inclination of the material presented during visuospatial processing. Moreover, the present results reinforce the view that a within-device effect may occur (rather than a device effect), depending on whether the device is used in a flat or a tilted position, as previously suggested by Salmerón et al. (2023).

Because prior work has not directly examined whether inclination and type of support jointly modulate global and local processing, the present findings provide the first indication that global and local interference may differ in their sensitivity to display inclination. In the tilted condition, participants showed stronger global interference, whereas in the flat condition local interference was more pronounced. Changes in display orientation are known to influence perceived verticality and the alignment of reference frames (Bagust et al., 2013; Goodenough et al., 1979; Morgan et al., 2015), which may in turn modulate how attention is distributed across hierarchical levels. In our study, tilt appears to favor a more classical global-precedence effect, whereas a flat orientation reduces the asymmetry between global and local interference, possibly by facilitating access to local information or reducing the salience of the global frame. Although not mutually exclusive, these hypotheses require further experimental testing to be disentangled. Interestingly, we did not observe any main effect or interaction involving the medium (paper vs. screen). This absence of a medium effect aligns with the small and context-dependent differences reported in studies of reading performance, which tend to emerge under specific conditions such as long expository texts, time pressure, or scrolling, rather than at visuo-attentional stages (Clinton, 2019; Delgado et al., 2018; Salmerón et al., 2023). Under the conditions implemented here, inclination appears to exert a stronger influence on global and local interference effects than the nature of the medium itself. It is therefore possible that the way we use materials in daily life, and the way they are presented as early as the school years, may influence attentional processing, in our case, global–local visual processing. Indeed, the position of materials during common activities, such as adults reading a book or schoolchildren working at a desk, may shape visuo-attentional processing from early age. Childhood is a transitional period during which the ability to process global and local visual information develops with age (Poirel et al., 2008a), and this developmental period is also associated with learning school material typically presented in a flat position. It seems conceivable that this mode of presentation could promote greater sensitivity to local information in children, in line with findings showing that children under 6 years of age exhibit a local precedence effect (see Poirel et al., 2008b, 2011, 2012). Although this assumption needs to be confirmed by future studies involving children, this habituation effect may have a potential impact on how children process visual information when it is presented flat than tilted. Using the flat position for reading in daily life, and beginning this practice during childhood, may thus contribute to the persistence of a local-to-global interference effect in adults when material is presented flat. Consequently, this additional process modulates the GPE observed during natural scene observation, where visual input is mainly encountered in a “tilted” position. Finally, many psychology experiments present stimuli on a computer screen. This tilted position may have masked the potential local interference effect observed in the present study, which was not reported in previous work. Future studies should therefore consider the potential impact of medium orientation alongside the characteristics of the medium itself. In particular, in line with evidence showing variations in cognitive ability between paper and tablet (e.g., Mangen et al., 2019), the orientation of the material may also influence the findings reported in previous studies.

The present study nevertheless has several limitations. First, daily screen time, as well as daily reading habits, which could potentially influence attentional resources, were not considered. Second, although we controlled viewing distance and the physical size of stimuli as closely as possible, both on screen and on paper, and in both flat and tilted conditions, slight postural variations (head tilt, trunk position) may have altered the actual visual angle at which the stimuli were perceived. This may have contributed, at least in part, to the effects attributed to the tilt condition. Future work would benefit from precisely measuring and calibrating this angle, for instance with a headrest. Moreover, it would be useful to examine multiple tilt angles (rather than only 0 and 110°) to more accurately assess the effect of medium inclination on cognitive processes. Furthermore, the present study used a between-subjects design. Although the observed differences are attributed to the manipulated factors, we cannot fully rule out the possibility of unmeasured group differences. Future research should employ alternative designs with appropriate covariates to replicate these findings and more clearly isolate the specific contributions of medium and orientation to global–local processing. Future work should also examine several school-age groups to determine how global–local attentional processing develops under tilted and flat conditions. For example, a longitudinal follow-up would be particularly relevant for advancing research on this topic.

## 5 Conclusion

In conclusion, the present experiment did not reveal differences between paper and screens during global/local processing, but it does suggest for the first time a potential effect of inclination on the global precedence phenomenon. The inclination factor should therefore be taken into account in future studies on digital media in order to provide a more fine-grained understanding of how humans process the visual world and how factors such as inclination influence cognitive processes more broadly.

## Data Availability

The raw data supporting the conclusions of this article will be made available by the authors, without undue reservation.

## References

[ref9001] Ackerman R. Lauterman T. (2012). Taking reading comprehension exams on screen or on paper? A metacognitive analysis of learning texts under time pressure. Comput. Hum. Behav. 28, 1816–1828. doi: 10.1016/j.chb.2012.04.023

[ref1] Albin T. J. McLoone H. E. (2014). The effect of tablet tilt angle on users' preferences, postures, and performance. Work 47, 207–211. doi: 10.3233/wor-131670, 24004729

[ref2] Bagust J. Docherty S. Razzak R. A. (2013). Rod and frame alignment times increase when the frame is tilted. Psychol. Behav. Sci. 2, 66–72. doi: 10.11648/j.pbs.20130202.17

[ref3] Bao H. W. S. (2023). *BruceR: broadly useful Covenient and efficient R Fuctions (R package version 2023.9)*. Available online at: https://cran.r-project.org/package=bruceR.

[ref4] Bar M. (2004). Visual objects in context. Nat. Rev. Neurosci. 5, 617–629. doi: 10.1038/nrn1476 15263892

[ref5] Beaucousin V. Cassotti M. Simon G. Pineau A. Kostova M. Houdé O. . (2011). ERP evidence of a meaningfulness impact on visual global/local processing: when meaning captures attention. Neuropsychologia 49, 1258–1266. doi: 10.1016/j.neuropsychologia.2011.01.039, 21281654

[ref6] Beyens I. Valkenburg P. M. Piotrowski J. T. (2018). Screen media use and ADHD-related behaviors: four decades of research. Proc. Natl. Acad. Sci. U. S. A. 115, 9875–9881. doi: 10.1073/PNAS.1611611114, 30275318 PMC6176582

[ref7] Bioulac S. Arfi L. Bouvard M. P. (2008). Attention deficit/hyperactivity disorder and video games: a comparative study of hyperactive and control children. Eur. Psychiatry 23, 134–141. doi: 10.1016/J.EURPSY.2007.11.002, 18206354

[ref8] Bosse M.-L. Valdois S. (2009). Influence of the visual attention span on child reading performance: a cross-sectional study. J. Res. Read. 32, 230–253. doi: 10.1111/j.1467-9817.2008.01387.x

[ref9] Bouhassoun S. Gerlach C. Borst G. Poirel N. (2022). Framing the area: an efficient approach for avoiding visual interference and optimising visual search in adolescents. Q. J. Exp. Psychol. 75, 2012–2022. doi: 10.1177/17470218211065011, 34812112

[ref9002] Brasel S. A. Gips J. (2011). Media Multitasking Behavior : Concurrent Television and Computer Usage. Cyberpsychol. Behav. Soc. Netw. 14, 527–534. doi: 10.1089/cyber.2010.0350 21381969 PMC3171998

[ref10] Bullier J. (2001). Integrated model of visual processing. Brain Res. Rev. 36, 96–107. doi: 10.1016/S0165-0173(01)00085-6, 11690606

[ref11] Camerini A.-L. Albanese E. Marciano L. (2022). The impact of screen time and green time on mental health in children and adolescents during the COVID-19 pandemic. Comput. Hum. Behav. Rep. 7:204. doi: 10.1016/j.chbr.2022.100204, 35611352 PMC9121633

[ref12] Cardoso-Leite P. Bavelier D. (2018). Video game play, attention, and learning: how to shape the development of attention and influence learning? Curr Opin Neurol 27, 185–191. doi: 10.1097/WCO.0000000000000077, 24553464

[ref13] Chan P. A. Rabinowitz T. (2006). A cross-sectional analysis of video games and attention deficit hyperactivity disorder symptoms in adolescents. Ann. General Psychiatry 5, 1–10. doi: 10.1186/1744-859X-5-16, 17059614 PMC1635698

[ref14] Choi E. Shin S. H. Ryu J. K. Jung K. I. Kim S. Y. Park M. H. (2020). Commercial video games and cognitive functions: video game genres and modulating factors of cognitive enhancement. Behav. Brain Funct. 16, 1–14. doi: 10.1186/S12993-020-0165-Z, 32014027 PMC6996164

[ref15] Clinton V. (2019). Reading from paper compared to screens: a systematic review and meta-analysis. J. Res. Read. 42, 288–325. doi: 10.1111/1467-9817.12269

[ref16] Cohen J. (2013). Statistical power analysis for the behavioral sciences. Abingdon: Routledge.

[ref17] Davies C. A. Vandelanotte C. Duncan M. J. van Uffelen J. G. Z. (2012). Associations of physical activity and screen-time on health related quality of life in adults. Prev. Med. 55, 46–49. doi: 10.1016/J.YPMED.2012.05.003, 22588226

[ref18] Delgado P. Salmerón L. (2022). Cognitive effort in text processing and reading comprehension in print and on tablet: an eye-tracking study. Discourse Process. 59, 237–274. doi: 10.1080/0163853X.2022.2030157

[ref19] Delgado P. Vargas C. Ackerman R. Salmerón L. (2018). Don’t throw away your printed books: a meta-analysis on the effects of reading media on reading comprehension. Educ. Res. Rev. 25, 23–38. doi: 10.1016/j.edurev.2018.09.003

[ref20] Domoff S. E. Borgen A. L. Foley R. P. Maffett A. (2019). Excessive use of mobile devices and children's physical health. Hum. Behav. Emerg. Technol. 1, 169–175. doi: 10.1002/hbe2.145

[ref21] Faul F. Erdfelder E. Lang A.-G. Buchner A. (2007). G*power 3: a flexible statistical power analysis program for the social, behavioral, and biomedical sciences. Behav. Res. Methods 39, 175–191. doi: 10.3758/BF03193146, 17695343

[ref22] Fredrickson B. Branigan C. (2005). Positive emotions broaden the scope of attention and thought action repertories. Cognit. Emot. 19, 313–332. doi: 10.1080/02699930441000238, 21852891 PMC3156609

[ref23] Gentile D. A. Swing E. L. Lim C. G. Khoo A. (2012). Video game playing, attention problems, and impulsiveness: evidence of bidirectional causality. Psychol. Pop. Media Cult. 1, 62–70. doi: 10.1037/A0026969

[ref24] Gerlach C. Poirel N. (2018). Navon’s classical paradigm concerning local and global processing relates systematically to visual object classification performance. Sci. Rep. 8:324. doi: 10.1038/s41598-017-18664-5, 29321634 PMC5762637

[ref25] Gerlach C. Poirel N. (2020). Who's got the global advantage? Visual field differences in processing of global and local shape. Cognition 195:104131. doi: 10.1016/j.cognition.2019.104131, 31731118

[ref26] Goodenough D. R. Oltman P. K. Sigman E. Rosso J. Mertz H. (1979). Orientation contrast effects in the rod-and-frame test. Percept. Psychophys. 25, 419–424. doi: 10.3758/BF03199851, 461103

[ref27] Green C. S. Bavelier D. (2003). Action video game modifies visual selective attention. Nature 423, 534–537. doi: 10.1038/nature01647 12774121

[ref28] Guellai B. Somogyi E. Esseily R. Chopin A. (2022). Effects of screen exposure on young children’s cognitive development: a review. Front. Psychol. 13:923370. doi: 10.3389/fpsyg.2022.923370, 36059724 PMC9431368

[ref29] Halamish V. Elbaz E. (2020). Children’s reading comprehension and metacomprehension on screen versus on paper. Comput. Educ. 145:103737. doi: 10.1016/J.COMPEDU.2019.103737

[ref30] Hegdé J. (2008). Time course of visual perception: coarse-to-fine processing and beyond. Prog. Neurobiol. 84, 405–439. doi: 10.1016/J.PNEUROBIO.2007.09.001, 17976895

[ref31] Kauffmann L. Ramanoël S. Peyrin C. (2014). The neural bases of spatial frequency processing during scene perception. Front. Integr. Neurosci. 8:87047. doi: 10.3389/FNINT.2014.00037 PMC4019851 24847226

[ref32] Krakowski C. S. Borst G. Pineau A. Houdé O. Poirel N. (2015). You can detect the trees as well as the forest when adding the leaves: evidence from visual search tasks containing three-level hierarchical stimuli. Acta Psychol. 157, 131–143. doi: 10.1016/j.actpsy.2015.03.001, 25796055

[ref33] Krakowski C. S. Poirel N. Vidal J. Roëll M. Pineau A. Borst G. . (2016). The forest, the trees and the leaves: differences of processing across development. Dev. Psychol. 52, 1262–1272. doi: 10.1037/dev0000138, 27455187

[ref34] Madigan S. Browne D. Racine N. Mori C. Tough S. (2019). Association between screen time and children’s performance on a developmental screening test. JAMA Pediatr. 173, 244–250. doi: 10.1001/JAMAPEDIATRICS.2018.5056, 30688984 PMC6439882

[ref35] Madigan S. McArthur B. A. Anhorn C. Eirich R. Christakis D. A. (2020). Associations between screen use and child language skills: a systematic review and meta-analysis. JAMA Pediatr. 174, 665–675. doi: 10.1001/jamapediatrics.2020.0327, 32202633 PMC7091394

[ref36] Mangen A. Olivier G. Velay J. L. (2019). Comparing comprehension of a long text read in print book and on kindle: where in the text and when in the story? Front. Psychol. 10:426051. doi: 10.3389/FPSYG.2019.00038 PMC6384527 30828309

[ref37] Mangen A. Walgermo B. R. Brønnick K. (2013). Reading linear texts on paper versus computer screen: effects on reading comprehension. Int. J. Educ. Res. 58, 61–68. doi: 10.1016/j.ijer.2012.12.002

[ref38] Margolin S. J. Driscoll C. Toland M. J. Kegler J. L. (2013). E-readers, computer screens, or paper: does reading comprehension change across media platforms? Appl. Cogn. Psychol. 27, 512–519. doi: 10.1002/ACP.2930

[ref39] Martinez L. Gimenes M. Lambert E. (2023). Video games and board games: effects of playing practice on cognition. PLoS One 18:e0283654. doi: 10.1371/journal.pone.0283654, 36972271 PMC10042352

[ref40] Masi L. Abadie P. Herba C. Emond M. Gingras M. P. Amor L. B. (2021). Video games in ADHD and non-ADHD children: modalities of use and association with ADHD symptoms. Front. Pediatr. 9:632272. doi: 10.3389/FPED.2021.632272, 33777866 PMC7994285

[ref41] McArthur B. A. Tough S. Madigan S. (2022). Screen time and developmental and behavioral outcomes for preschool children. Pediatr. Res. 91, 1616–1621. doi: 10.1038/s41390-021-01572-w, 34012028

[ref42] Morgan M. Grant S. Melmoth D. Solomon J. A. (2015). Tilted frames of reference have similar effects on the perception of gravitational vertical and the planning of vertical saccadic eye movements. Exp. Brain Res. 233, 2115–2125. doi: 10.1007/s00221-015-4282-0, 25921228 PMC4464849

[ref43] Muppalla S. K. Vuppalapati S. Reddy Pulliahgaru A. Sreenivasulu H. (2023). Effects of excessive screen time on child development: an updated review and strategies for management. Cureus 15:e40608. doi: 10.7759/cureus.40608, 37476119 PMC10353947

[ref44] Navon D. (1977). Forest before trees: the precedence of global features in visual perception. Cogn. Psychol. 9, 353–383. doi: 10.1016/0010-0285(77)90012-3

[ref45] Navon D. (1981). The forest revisited: more on global precedence. Psychol. Res. 43, 1–32. doi: 10.1007/BF00309635

[ref46] Ozerbas M. A. Erdogan B. H. (2016). The effect of the digital classroom on academic success and online technologies self-efficacy. Educ. Technol. Soc. 19, 203–212. doi: 10.2307/jeductechsoci.19.4.203

[ref47] Peñuelas-Calvo I. Jiang-Lin L. K. Girela-Serrano B. Delgado-Gomez D. Navarro-Jimenez R. Baca-Garcia E. . (2022). Video games for the assessment and treatment of attention-deficit/hyperactivity disorder: a systematic review. Eur. Child Adolesc. Psychiatry 31, 5–20. doi: 10.1007/S00787-020-01557-W, 32424511

[ref48] Poirel N. Cassotti M. Beaucousin V. Pineau A. Houdé O. (2012). Pleasant emotional induction broadens the visual world of young children. Cognit. Emot. 26, 186–191. doi: 10.1080/02699931.2011.589430, 21824012

[ref49] Poirel N. Krakowski C. S. Sayah S. Pineau A. Houdé O. Borst G. (2014). Do you want to see the tree? Ignore the forest: a negative priming study of local-global processing. Exp. Psychol. 61, 205–214. doi: 10.1027/1618-3169/A000240, 24217136

[ref50] Poirel N. Mellet E. Houdé O. Pineau A. (2008a). First came the trees, then the forest: developmental changes during childhood in the processing of visual local-global patterns according to the meaningfulness of the stimuli. Dev. Psychol. 44, 245–253. doi: 10.1037/0012-1649.44.1.245, 18194023

[ref51] Poirel N. Pineau A. Jobard G. Mellet E. (2008b). Seeing the forest before the trees depends on individual field-dependency characteristics. Exp. Psychol. 55, 328–333. doi: 10.1027/1618-3169.55.5.328, 25116300

[ref52] Poirel N. Simon G. Cassotti M. Leroux G. Perchey G. Lanoë C. . (2011). The shift from local to global visual processing in 6-year-old children is associated with Grey matter loss. PLoS One 6:e20879. doi: 10.1371/JOURNAL.PONE.0020879, 21687636 PMC3110822

[ref54] Radesky J. S. Christakis D. A. (2016). Increased screen time: implications for early childhood development and behavior. Pediatr. Clin. N. Am. 63, 827–839. doi: 10.1016/j.pcl.2016.06.006, 27565361

[ref53] R Core Team. (2022). *R: a language and environment for statistical computing*. R Foundation for Statistical Computing. Available online at: https://www.R-project.org/.

[ref55] Rockinson-Szapkiw A. J. Courduff J. Carter K. Bennett D. (2013). Electronic versus traditional print textbooks: a comparison study on the influence of university students’ learning. Comput. Educ. 63, 259–266. doi: 10.1016/j.compedu.2012.11.022

[ref9003] Roux F. Ceccaldi M. (2001). Does aging affect the allocation of visual attention in global and local information processing? Brain Cognit. 46, 383–396. doi: 10.1006/brcg.2001.1296 11487288

[ref56] Russell W. D. Newton M. (2008). Short-term psychological effects of interactive video game technology exercise on mood and attention. Educ. Technol. Soc. 11, 294–308.

[ref57] Salmerón L. Altamura L. Delgado P. Karagiorgi A. Vargas C. (2023). Reading comprehension on handheld devices versus on paper: a narrative review and meta-analysis of the medium effect and its moderators. J. Educ. Psychol. 116, 153–172. doi: 10.1037/EDU0000830, 41340023

[ref58] Schubert T. Finke K. Redel P. Kluckow S. Müller H. Strobach T. (2015). Video game experience and its influence on visual attention parameters: an investigation using the framework of the theory of visual attention (TVA). Acta Psychol. 157, 200–214. doi: 10.1016/j.actpsy.2015.03.005, 25834984

[ref59] Serences J. T. Kastner S. (2014). “A multi-level account of selective attention” in The Oxford handbook of attention. eds. Nobre K. Kastner S., vol. 1 (Oxford, UK: Oxford University Press), 76–104.

[ref60] Sparrow B. Liu J. Wegner D. M. (2011). Google effects on memory: cognitive consequences of having information at our fingertips. Science 333, 776–778. doi: 10.1126/SCIENCE.1207745, 21764755

[ref61] Støle H. Mangen A. Schwippert K. (2020). Assessing children's reading comprehension on paper and screen: a mode-effect study. Comput. Educ. 151:103861. doi: 10.1016/j.compedu.2020.103861

[ref62] Swing E. L. Gentile D. A. Anderson C. A. Walsh D. A. (2010). Television and video game exposure and the development of attention problems. Pediatrics 126, 214–221. doi: 10.1542/PEDS.2009-1508, 20603258

[ref63] Tang S. Werner-Seidler A. Torok M. Mackinnon A. J. Christensen H. (2021). The relationship between screen time and mental health in young people: a systematic review of longitudinal studies. Clin. Psychol. Rev. 86:102021. doi: 10.1016/J.CPR.2021.102021, 33798997

[ref64] Valdois S. Bosse M.-L. Tainturier M.-J. (2019). Visual attention modulates reading acquisition. Vis. Res. 165, 152–161. doi: 10.1016/j.visres.2019.10.011, 31751900

[ref65] Vizcaino M. Buman M. Desroches T. Wharton C. (2020). From TVs to tablets: the relation between device-specific screen time and health-related behaviors and characteristics. BMC Public Health 20, 1–10. doi: 10.1186/S12889-020-09410-0, 32847553 PMC7447608

[ref66] Wickham H. (2016). ggplot2: Elegant graphics for data analysis. Berlin: Springer-Verlag.

[ref67] Wilms I. L. Petersen A. Vangkilde S. (2013). Intensive video gaming improves encoding speed to visual short-term memory in young male adults. Acta Psychol. 142, 108–118. doi: 10.1016/j.actpsy.2012.11.003, 23261420

[ref68] Xu B. Chen G. Huang R. Sun Y. (2017). *The effectiveness of media platforms on reading comprehension: a meta-analysis*. Conference: The 25th International Conference on Computers in Education.At: Christchurch, New Zealand.

[ref69] Young J. G. Trudeau M. Odell D. Marinelli K. Dennerlein J. T. (2012). Touch-screen tablet user configurations and case-supported tilt affect head and neck flexion angles. Work 41, 81–91. doi: 10.3233/WOR-2012-1337, 22246308

